# Targeting Autophagy for Cancer Treatment and Tumor Chemosensitization

**DOI:** 10.3390/cancers11101599

**Published:** 2019-10-19

**Authors:** Marta Pérez-Hernández, Alain Arias, David Martínez-García, Ricardo Pérez-Tomás, Roberto Quesada, Vanessa Soto-Cerrato

**Affiliations:** 1 Department of Pathology and Experimental Therapeutics, Faculty of Medicine and Health Sciences, Universitat de Barcelona, 08905 Barcelona, Spain; martaperezh@ub.edu (M.P.-H.); alain.arias@ufrontera.cl (A.A.); david.martinez@ub.edu (D.M.-G.); rperez@ub.edu (R.P.-T.); 2 Oncobell Program, Institut d’Investigació Biomèdica de Bellvitge (IDIBELL), L’Hospitalet de Llobregat, 08908 Barcelona, Spain; 3 Department of Integral Adult Dentistry, Research Centre for Dental Sciences (CICO), Universidad de La Frontera, Temuco 4811230, Chile; 4 Research Group of Health Sciences, Faculty of Health Sciences, Universidad Adventista de Chile, Chillán 3780000, Chile; 5 Department of Chemistry, Universidad de Burgos, 09001 Burgos, Spain; rquesada@ubu.es

**Keywords:** autophagy, anticancer therapy, autophagy inhibitors, autophagic cell death, chemoresistance, chemosensitization

## Abstract

Autophagy is a tightly regulated catabolic process that facilitates nutrient recycling from damaged organelles and other cellular components through lysosomal degradation. Deregulation of this process has been associated with the development of several pathophysiological processes, such as cancer and neurodegenerative diseases. In cancer, autophagy has opposing roles, being either cytoprotective or cytotoxic. Thus, deciphering the role of autophagy in each tumor context is crucial. Moreover, autophagy has been shown to contribute to chemoresistance in some patients. In this regard, autophagy modulation has recently emerged as a promising therapeutic strategy for the treatment and chemosensitization of tumors, and has already demonstrated positive clinical results in patients. In this review, the dual role of autophagy during carcinogenesis is discussed and current therapeutic strategies aimed at targeting autophagy for the treatment of cancer, both under preclinical and clinical development, are presented. The use of autophagy modulators in combination therapies, in order to overcome drug resistance during cancer treatment, is also discussed as well as the potential challenges and limitations for the use of these novel therapeutic strategies in the clinic.

## 1. Introduction

Cellular homeostasis is crucial for cell survival and refers to all processes involved in the maintenance of an internal steady state at the level of the cell. Autophagy is one of the main catabolic mechanisms that contributes to cellular homeostasis, through the degradation and recycling of cytoplasmic components and organelles in the lysosomes [1,2]. This process confers the ability to adapt to environmental stresses, preventing cellular damage, and promoting cell survival, even in starving conditions, thus having a main physiologic cytoprotective role. It is a process tightly regulated and its dysfunction has been related to several pathologies, such as neurodegeneration, cancer, or aging [3]. Hence, autophagy modulation is emerging as a promising new therapeutic strategy to treat these malignancies [4]. Indeed, more than 120 clinical trials related to the process of autophagy were initiated to date. The majority of those target autophagy for cancer treatment, already showing promising results, for instance, using chloroquine or hydroxychloroquine as single agents or in combination therapies [5,6]. Nevertheless, the role of autophagy in cancer is somewhat controversial. Cytotoxic or cytoprotective roles have been reported depending on the cellular context [7]. Therefore, the deep understanding of autophagy regulation and the identification of its role in each cellular context is crucial for the selection of an appropriate therapeutic intervention involving autophagy modulation in cancer.

In this review, the molecular mechanisms that regulate autophagy and the dual role of autophagy in cancer are presented and discussed. Moreover, current strategies targeting autophagy for cancer treatment are summarized, highlighting combination therapies involving autophagy modulators that can sensitize cancer cells to conventional therapies, thus being able to overcome chemoresistance.

## 2. Autophagy Process and Regulation

The term autophagy encompasses diverse cellular processes that are characterized by lysosomal degradation of cytoplasmic material. Three different types of autophagy have been described: Microautophagy, chaperone mediated autophagy (CMA), and macroautophagy [8]. Microautophagy consists of direct engulfment, via lysosomal membrane invagination, of cytoplasmic material that has to be degraded [9]. In mammals, this process also occurs in late endosomes, which is known as endosomal microautophagy [10]. CMA involves the targeting of specific proteins by chaperones and their delivery to lysosomes for their subsequent degradation [11]. Finally, macroautophagy is a highly conserved process involving the formation of autophagosomes, double membrane vesicles that engulf cytoplasmic components and fuse with the lysosomes for their content degradation [2]. A specific type of macroautophagy is called mitophagy, which consists of the selective degradation of damaged mitochondria by autophagy, promoting their turnover, and preventing the accumulation of dysfunctional mitochondria [12].

**Figure 1:**
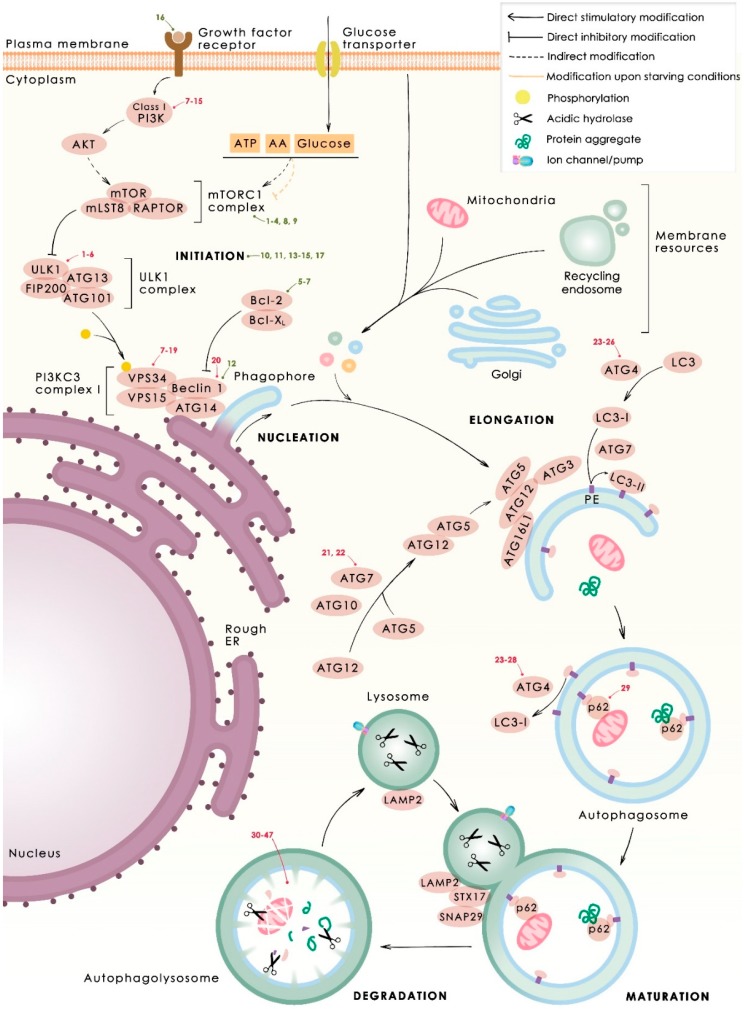
Mechanism of autophagy. The phases of the process of autophagy (nucleation, elongation, maturation, and degradation), with the main proteins that participate in each one, are depicted. Autophagy activators (**green**) and inhibitors (**red**) are marked where they interfere with the autophagy process. Numbers correspond to those compounds listed in Table 1 and Table 2, respectively.

Macroautophagy (here on called autophagy) has multiple steps, including the initiation, nucleation, elongation, maturation, and vesicle content degradation (Figure 1). This process is tightly regulated at the molecular level by a family of proteins called autophagy-related proteins (ATGs) [13]. The initiation step can be triggered through diverse stresses, such as growth factors or nutrient deprivation, hypoxia, oxidative stress, and protein aggregation, among others [4]. Under starvation, low levels of glucose, amino acids, or growth factors provoke the activation of Unc-51-like kinase 1 (ULK1) complex (consisting of ULK1, ATG13, RB1-inducible coiled-coil protein 1 (FIP200) and ATG101)) through the inhibition of the mammalian target of rapamycin complex 1 (mTORC1) [14,15]. Similarly, low levels of ATP activate AMP-activated protein kinase (AMPK), inducing mTORC1 inhibition and subsequent ULK1 activation [16]. Once ULK1 complex is activated (initiation step), it induces the phosphorylation and activation of the catalytic subunit of vacuolar protein sorting 34 (VPS34), a class III phosphatidylinositol 3-kinase (PI3KC3) that forms a complex with VPS15, Beclin-1, and ATG14. During the vesicle nucleation step, Beclin-1 acts as a scaffold for the rest of the components and VPS34 converts phosphatidylinositol (PI) to PI-3-phosphate (PI3P) giving rise to the phagophore, the initial portion of double membrane that will enclose the cytoplasmic material. This process takes place in the endoplasmic reticulum by the anchoring of the PI3KC3 complex through ATG14 protein to a region called omegasome [17]. After phagophore formation, ATG7 and ATG10 mediate ATG12 conjugation to ATG5, which form a complex with ATG16L1 that attaches to to the autophagosome membrane during vesicle elongation. At the same time, microtubule-associated protein light chain 3 (LC3, also named ATG8) is cleaved by the protease ATG4 to form LC3-I, then it is activated by ATG7 and finally ATG3, at the vesicle membrane, promoting the conjugation of LC3-I (or γ-aminobutiric acid receptor-associated proteins (GABARAPs)) with membrane-resident phosphatidylethanolamine (PE) to form LC3-II that will permit cargo recognition and vesicle elongation and fusion [18,19]. Apart from the endoplasmic reticulum, other compartments also act as membrane donors, such as Golgi, recycling endosomes, mitochondria, or plasma membrane [20,21]. After vesicle expansion and sealing, the autophagosome maturates and fuses with the lysosome, resulting in an autophagolysosome. Several soluble N-ethylmaleimide-sensitive factor activating protein receptor (SNARE) proteins, such as syntaxin 17 (STX17) and synaptosomal-associated protein 29 (SNAP29) or the lysosome-associated membrane protein 2 (LAMP2) participate in the fusion of autophagosomes with lysosomes [22,23]. Finally, acidic hydrolases coming from the lysosome degrade the autophagic cargo and the resulting products, such as amino acids or fatty acids, are finally recycled to the cytosol.

## 3. Dual Role of Autophagy in Cancer

Autophagy has opposing roles in cancer, preventing tumor initiation in healthy tissues, but favoring cancer progression once the tumor is formed [4]. Before the onset of carcinogenesis, the cytoprotective role of autophagy has been described to mainly act as a tumor suppressor mechanism, mitigating metabolic stress and genome instability that may cause tumor initiation [24,25]. Indeed, loss of function of ATGs, such as Beclin-1, has been associated with increased risk of cancer [26,27]. For instance, the suppression of ATG proteins in mice has been shown to provoke multiple benign tumors in liver [28]. However, once the primary tumor is formed, the role of autophagy in cancer cells varies, being cytotoxic or cytoprotective depending on the cellular context [7]. Therefore, it is crucial to understand the dual role of autophagy in cancer cells for the selection of a successful therapeutic strategy targeting autophagy.

On one hand, some tumors can undergo autophagic cell death (ACD) upon the induction of autophagy by some anticancer drugs, acting as a cytotoxic process [29,30]. ACD frequently occurs in cancer cells lacking functional apoptotic machinery, such as p53-deficient cancer cells [31]. Hence, autophagy induction may be an appropriate therapeutic strategy for this type of tumors. Likewise, autophagy activation has also been described as beneficial when combined with therapies that induce immunogenic cell death, since cells dying from autophagy release certain molecules, damage-associated molecular pattern molecules (DAMPs), which recruit immune effectors enhancing the therapeutic response [8,32,33].

On the other hand, some tumors trigger autophagy as a response to mitigate the cellular stress induced by an anticancer drug. In this case, inhibition of autophagy sensitizes cancer cells to therapy, enhancing the cytotoxic effects induced by chemotherapeutic agents [34,35,36,37]. Indeed, autophagy has been described as a pro-survival mechanism present in most advanced tumors, facilitating tumor adaptation to different stresses, such as hypoxia or nutrient deprivation, thus mediating tumor progression [38,39]. Accordingly, many types of advanced cancers show higher basal autophagic activity than normal tissues, and some have been described as “autophagy-dependent” tumors, such as pancreatic cancer or activated Ras tumors [40,41]. This basal autophagy also facilitates cancer cell adaptation to therapy-induced stresses, provoking therapy resistance in these tumors, which is one of the major challenges in the clinic. Moreover, autophagy has also been related to the survival of dormant cancer cells and metastatic tumor recurrence [42]. Hence, the inhibition of autophagy may be an appropriate therapeutic strategy to treat these tumors.

## 4. Therapeutic Strategies Targeting Autophagy

Modulation of autophagy has emerged as a promising therapeutic option for cancer treatment. Due to the dual role of autophagy in cancer cells, activators as well as inhibitors have been described as feasible chemotherapeutic agents.

In this section, we compiled different therapeutic interventions targeting autophagy, either for its stimulation or for its inhibition (Figure 1).

### 4.1. Autophagy Stimulation for Cancer Treatment

Induction of ACD has become an interesting alternative to overcome resistance to apoptosis and to exploit a caspase independent cell death for cancer treatment. In the following sections, compounds for which the mechanism of action is based on stimulating autophagy are described (Table 1). 

#### 4.1.1. mTOR Inhibitors

The mTOR is a protein kinase that participates in multiple cellular processes such as cell growth, survival, metabolism, and immunity. Thus, mTOR regulates several cellular mechanisms including cell cycle, apoptosis, and autophagy [74], inhibiting the initiation of the latter process [75]. Rapamycin (sirolimus), a secondary metabolite isolated from *Streptomyces hygroscopicus,* showed potent antifungal, antitumor, and immunosuppressive properties [76,77]. Rapamycin and its semi-synthetic analogues, known as rapalogs, are allosteric selective inhibitors of mTORC1 affecting downstream targets, including the activation of autophagy [78,79]. However, their efficacy inhibiting tumor growth is limited due to lack of inhibition of mTORC2 and other compensatory signaling pathways that promote cell survival [80].

Rapamycin has shown to inhibit proliferation and induce ACD in murine sarcoma [43], neuroblastoma [44], lung cancer [45], and osteosarcoma [46]. Conversely, the rapalog temsirolimus or cell cycle inhibitor-779 (CCI779), has shown to inhibit tumor growth in vitro in adenoid cystic carcinoma [47] but has also shown to stimulate autophagy as a pro-survival mechanism in renal-cell carcinoma [48]. Additionally, everolimus (or RAD001), a derivative rapalog developed for oral administration, has shown to induce cell cycle arrest through autophagy-mediated degradation of cyclin D1 in breast cancer cells [49], but promotes autophagy in aromatase inhibitor-resistant breast cancer cells as a mechanism of resistance [50].

Other types of mTOR inhibitors are compounds that compete with ATP, impeding phosphorylation of its target proteins, resulting in a more efficient inhibition of mTOR [81]. Among them, AZD8055 inhibits both mTOR complexes and has shown to inhibit tumor growth [51] and induce ACD in hepatocellular carcinoma cell lines [52], but it is also capable of limiting tumor growth through induction of apoptosis and cell cycle arrest [82]. Taken together these findings suggest that mTOR inhibitors may act through different mechanisms to induce cell death in a tumor context dependent manner, which makes them suitable for combined therapies to overcome cancer cell resistance [83]. 

#### 4.1.2. BH3 Mimetics

BH3 (Bcl-2 homology 3) mimetics are a group of small molecules that mimic interactions of BH3-only proteins [84], which are a sub-group of pro-apoptotic proteins in the Bcl-2 family [85]. In general, BH3 mimetics may stimulate autophagy by liberating Beclin-1 from Bcl2 and Bcl-X_L_ inhibition [85,86].

Gossypol is a BH3 mimetic isolated from cotton that has a high affinity for Bcl-2, Bcl-X_L_, Mcl-1, and Bcl-w [85]. Its orally available enantiomeric form (-)-gossypol (AT-101) has shown to induce ACD in malignant glioma [56], but the induced autophagy has also been accompanied by apoptosis in head and neck squamous cell carcinoma [53], malignant mesothelioma [54], and colon cancer cells [55]. Obatoclax (GX15-070) is another BH3 mimetic that has shown autophagic-mediated necroptosis in oral squamous cell carcinoma [57], rhabdomyosarcoma cells [58], and acute lymphoblastic leukemia cells [59]. Moreover, obatoclax induced autophagy in adenoid cystic carcinoma [87] and Beclin-1 independent autophagy inhibition in colorectal cancer cells [88]. Finally, ABT-737 has shown effectivity in vitro for hepatocellular carcinoma cells in a Beclin-1-dependent autophagy manner [60].

#### 4.1.3. Cannabinoids

Cannabinoids are a group of more than 60 lipophilic ligands for specific cell-surface cannabinoid receptors (CB_1 y 2_) present in the plant cannabis sativa, with Δ^9^-Tetrahydrocannabinol (THC) being the main psychoactive compound [89]. Cannabinoids have shown potent anticancer effects related to autophagy, but they have also shown cytoprotective effects depending on cell type and cannabinoid used [90]. THC has shown to activate non-canonical autophagy-mediated apoptosis in melanoma cells [61] and induce ACD in glioma cells through mTORC1 inhibition and autolysosome permeabilization with the consequent release of cathepsins and posterior induction of apoptosis [62,63]. JWH-015 is a synthetic cannabinoid CB_2_ receptor-selective agonist that has shown to inhibit tumor growth through an autophagy-dependent mechanism in hepatocellular carcinoma cells and in vivo models through inhibition of Akt/mTORC1-pathway via AMPK activation [64].

#### 4.1.4. Histone Deacetylase Inhibitors (HDACIs)

The HDAC family includes four classes (I-IV) of transcriptional repressors that alter the structure of chromatin (via deacetylation) [91] and have been studied as anticancer compounds based on their potential to regulate gene expression [92]. Although apoptosis has been referred to as the main route for HDACIs-induced cancer cell death, autophagy stimulation has also been implicated, being the inactivation of PI3K/Akt/mTOR signaling the most described pathway [93].

Suberoylanilide hydroxamic acid (SAHA, Vorinostat) (a pan HDAC inhibitor) was the first HDACI approved by the FDA for the treatment of cutaneous T-cell lymphoma [94] that has shown to inhibit tumor growth through autophagy stimulation via activation of Cathepsin B in breast cancer cells in vitro [65]. Finally, MHY2256 (a synthetic class III HDAC inhibitor) has shown to induce ACD, cell cycle arrest and apoptosis in endometrial cancer cells in both in vitro and in vivo [66].

#### 4.1.5. Natural Products

Some natural compounds have shown promising anticancer activities based on autophagy stimulation. Betulinic acid is a pentacyclic triterpenoid derived from widespread plants that has shown to induce ACD in multiple myeloma cells with high levels of Bcl-2 expression. This derivative acts as an attenuator for mitochondrial-mediated apoptosis, promoting ACD by inducing Beclin-1 phosphorylation [67]. Resveratrol, a polyphenol compound widely found in plants, has been shown to inhibit cell proliferation in breast cancer stem-like cells via suppressing the Wnt/b-catenin signaling pathway [68]. This pathway, which regulates critical genes in tissue development and homeostasis, is aberrantly activated in many cancers and its inhibition has been reported to be related with autophagy processes [68,95]. δ-Tocotrienol is one of the four isomers that comprises vitamin E that has shown cytotoxic effects against prostate cancer cells in vitro through autophagy activation via ER stress [69]. Curcumin is a major constituent of *Curcuma longa* (turmeric) that induces autophagy, which has been shown to elicit a dual role protecting or leading to cell death depending on the duration of the treatment and concentration used [70]. 

#### 4.1.6. Others

Other compounds have been reported to induce ACD in cancer. For example, lapatinib is a small molecule tyrosine kinase inhibitor, targeting epidermal growth factor receptors that is capable of inducing ACD in hepatocellular carcinoma [71] and in acute leukemia cell lines [72]. APO866 is an inhibitor of nicotinamide adenine dinucleotide (NAD) biosynthesis that has shown anticancer activity through induction of ACD in cells from hematological malignancies [73].

### 4.2. Autophagy Inhibition for Cancer Treatment

In several tumors, autophagy has a protective role; therefore, its inhibition could be an interesting approach for tumor treatment. There are several autophagy inhibitors that block the process of autophagy at different steps, which we detail below (Table 2).

#### 4.2.1. ULK Inhibitors

ULKs are a family of serine/threonine protein kinases that form complexes with multiple regulator units. The role of ULK1 is essential for the initiation of autophagy [15,160,161], however the role of ULK2 in autophagy seems to be cell type dependent [162]. Due to the homology between ULK1 and ULK2 [163], inhibitors of ULK1 also inhibit ULK2 [163]. ULK1 has been shown to be upregulated in several cancers, which correlated with poor prognosis and treatment resistance [99,164,165,166]. Inhibition of ULK1 has been shown to induce a decrease in tumor growth and induction of apoptosis [100,101]. This has led to the search for compounds that inhibit this kinase activity finding some molecules that compete with the ATP-binding site, such as compound 6 [96], MRT68921, and MRT67307 [97,98]. Besides them, SBI-0206965 is the most studied [102], which inhibits autophagy and induces apoptosis in neuroblastoma cell lines [100], non-small cell lung cancer (NSCLC) cells [101,102], and in clear cell renal carcinoma cells [99]. Moreover, it has also been reported to be a direct inhibitor of AMPK, which is a serine/threonine kinase that activates the ULK complex, among other roles [167]. Recently more ULK inhibitors, such as ULK100 and ULK101, have been described [103], which supports that the idea that blocking ULK1 may be a good strategy for cancer therapy. 

#### 4.2.2. Pan PI3K Inhibitors

The family of phosphoinositide 3-kinases (PI3Ks) is divided into three classes with different substrate preferences, which define their functions. The role of class II on autophagy is unclear. However, class I activates mTORC1 through the PI3K/Akt pathway and consequently inhibits autophagy, while class III (VPS34) activates autophagy [168]. PI3K pathways have been associated with cancer due to their participation in tumorigenic processes such as cell proliferation, survival, migration, and angiogenesis. Therefore, they are a good target for therapy development [169]. Most of the studied PI3K inhibitors are not selective for a specific class of PI3K, hence, they affect different cellular processes, not only autophagy, and consequently their effect cannot be only attributed to inhibition of autophagy. However, due to their therapeutic relevance, we describe briefly some of them below.

3-Methyladenine (3MA) was one of the first inhibitors of autophagy described [104]. It exerts a dual effect on autophagy. Under starving conditions it suppresses autophagy through PI3KC3 inhibition. However, in the presence of nutrients it promotes autophagy by inhibition of PI3KC1 [105]. Additionally, it has been reported that it reduces the expression of drug efflux transporters, overcoming taxol and doxorubicin resistance [106]. 3MA is effective at high concentrations, although presents solubility problems. In order to overcome this limitation some derivatives have been synthetized [107]. Wortmannin is a fungal metabolite that binds irreversibly to the catalytic site of PI3Ks [108,109]. LY294002 is a synthetic small molecule [110] with poor solubility and short half-life. A conjugate analog of LY294002, named SF1126, was designed to accumulate in integrin expressing tissues, improving LY294002 solubility and pharmacokinetic, favoring its accumulation in the tumor site and showing antitumor and antiangiogenic properties in mouse models [111,112]. Other non-selective Pan PI3K inhibitors are PI103 [113], KU55933, Gö6976 [114], and GSK1059615 [115,116,170].

#### 4.2.3. VPS34 (PI3KC3) Complex Inhibitors

VPS34 is a PI3KC3 that transforms PI to PI3P. VPS34 forms a complex with several subunits needed for its activation, such as VPS15 (also known as p150), ATG14, and Beclin-1. Autophagy can be blocked by inhibition of VPS34 activity; SAR405 is one compound of the (2S)-tetrahydropyrimido-pyrimidinones series with kinase inhibitor activity by strong competition for ATP site. However, it is highly selective for PI3KC3, compared to class I and II, and more than 200 protein kinases and 15 lipid kinases. SAR405 inhibits autophagy induced either by starvation or mTOR inhibition [113]. VPS34-IN1 is a bipyrimidinamine that inhibits PI3KC3 selectively, compared with more than 300 protein kinases analyzed [117]. Additionally, PIK-III, a bisaminopyrimidine, binds to a hydrophobic pocket unique in VPS34 that cannot be found in other related kinases [118]. Compound 31 is a small molecule selective against protein and other lipid kinases [119]. All these four inhibitors are selective for PI3KC3, but it should be noted that VPS34 can form different complexes with other subunits that lead to a different localization and function, participating also in vesicle trafficking [171]. Thus, inhibitors of VPS34 can also have an effect on endosomal trafficking, as the case of SAR405 that prevents the activity of both VPS34 complexes [113]. Therefore, it may also affect cellular secretion [172].

On the other hand, autophagy can be also inhibited blocking PI3KC3 complex formation; Spautin-1 indirectly inhibits the activity of VPS34 by proteosomal degradation of proteins that form VPS34 complexes through reduction of Beclin-1 deubiquitination mediated by USP10 and USP13 [120]. 

#### 4.2.4. ATG inhibitors

Membrane PI3P produced by VPS34 leads to the recruitment of PI3P-binding ATG proteins and additional factors, resulting in the formation of complexes that participate in the elongation of the phagophore. Inhibition of autophagy can be achieved by impeding the formation of these complexes.

ATG7 participates in the formation of the complex ATG12-ATG5 and the conjugation of PE to LC3 and GABARAP. Recently, some inhibitors of ATG7 (WO2018/089786) have been designed and it has extended the use of micro RNAs that target ATG7 gene such as miR-154 that inhibits blade cancer progression [121].

On the other hand, ATG4B cleaves LC3, activating it for its conjugation with PE [173] necessary for the expansion of the autophagosome and its recognition. Additionally, it participates in LC3-PE deconjugation, which is important for LC3 recycling and for the fusion of the autophagosome with the lysosome [174]. Therefore, ATG4B could be a good target to inhibit autophagy more selectively, thus, a large number of ATG4B possible inhibitors have been screened in the last years [175]. NSC185058 is a small compound that docked at the active site of ATG4B inhibiting not only autophagy but also the volume of the autophagosomes, which is accompanied by suppression of tumor growth in an osteosarcoma subcutaneous mouse model [122]. Tioconazole is an antifungal drug that binds to the active site of ATG4 blocking autophagy flux reducing cell viability and sensitizing tumor cells to doxorubicin in a xenograft mouse model [123]. Other ATG4B inhibitors that suppress autophagy in cell lines and in vivo inhibiting cell proliferation are UAMC-2526, a derivative of benzotropolones stable in plasma [124], and LV-320, a styrylquinoline [125].

It should be noticed that the roles of ATG4B in cancer are not well understood and some of the ATG4 inhibitors showed only inhibition in LC3-PE delipidation, but not in the autophagosome formation such as S130 [126] and FMK-9a [127,128,129]. Additionally, some studies are focused on the evaluation of different markers that may predict the effectiveness of those inhibitors [176]. For instance, ATG4B inhibition is effective only in Her-2 positive cells and not in those negative [177].

#### 4.2.5. Autophagosome Formation Inhibition

Verteporfin is a benzoporphyrin derivative used in the clinic in photodynamic therapy. Interestingly, it prevents autophagosome formation induced by glucose and serum deprivation, but not by mTOR inhibition [130]. One possible mechanism of action of verteporfin is the blockade of p62 oligomerization, a protein necessary for the sequestration of ubiquitinated targets into autophagosomes [178,179]. Additional to autophagy inhibition, verteporfin reduces [131,133] transcriptional co-activators that regulate the Hippo pathway, implicated in cell growth and stem cell function [180]. Verteporfin inhibits cell proliferation, angiogenesis, and migration, and induces apoptosis [181]. It inhibits autophagy in vivo but has no effect as a single agent in tumor growth. However, it moderately sensitizes tumor cells to cytotoxic agents [132].

#### 4.2.6. Lysosome Inhibitors

The last step in autophagy is the fusion of autophagosomes with lysosomes, whose hydrolases degrade the autophagosome content. The inhibition of autophagy at this point consists of the use of lysosomal inhibitors.

Chloroquine (CQ) and its analog hydroxychloroquine (HCQ) [136] are drugs used for the treatment of various diseases, such as malaria and more recently cancer [135]. They are weak bases and the unprotonated form of CQ/HCQ can diffuse through cell membranes and enter into organelles such as lysosomes, where the high concentration of H^+^ induces their protonation and consequently increases lysosomal pH [134]. Once CQ/HCQ are protonated, they are trapped in the lysosomes producing an increase of their volume, and inhibiting the activity of lysosomal enzymes.

CQ and HCQ are the only autophagy inhibitors approved for clinical use. Although short-term CQ/HCQ treatment has been considered safe, retinopathy has been reported produced by long-term treatment with HCQ in about 7.5% of patients [182] and cardiotoxicity [183]. The prevalence depends on the dosage and the duration of treatment [184]. This toxicity limitation, along with inconsistencies in the results obtained in the clinic, have led to the study of new and more potent autophagy inhibitors [185]. Thus, CQ analogs that exert more potent autophagy inhibitory activity have been synthetized. Lys05 is a dimeric analog of CQ that accumulates within acidic organelles, including lysosomes, more potently than HCQ [137]. DQ661, a dimeric quinacrine (DQ), not only inhibits lysosomal catabolism, including autophagy, but also targets palmitoyl-protein thioesterase-1, resulting in the inhibition of mTORC1 signaling. DQ661 has shown effects on tumor mouse models alone and it also overcame resistance to gemcitabine [139]. Another antimalaria compound found to inhibit autophagy with antitumoral properties is VATG-027 [140]. On the other hand, mefloquine is also accumulated in lysosomes disrupting autophagy, it induces apoptosis and inhibits multidrug resistance protein1 (MDR1) being effective in multidrug-resistant tumor cells [142]. Mefloquine sensitizes chronic myeloid leukemia (CML) cells derived from patients in chronic phase to TK inhibitors showing selectivity for stem/progenitor tumoral cells to normal cells [141].

CQ and its derivatives are not the only drugs that target lysosomes to inhibit autophagy; GLP (ganoderma lucidum polysaccharide) is a polysaccharide from the fungus *Ganoderma lucidium* with multiple antitumoral properties [143]. GLP induces apoptosis in cancer cell lines [145] and reduces tumor growth in mouse models [144]. It has recently been seen that GLP impairs autophagy flux by reduction of lysosome acidification and the accumulation of autophagosomes has suggested to be the cause of apoptosis induction [144]. Bafilomycin A (BafA) is a vacuolar-H^+^ ATPase inhibitor that disrupts the acidification of lysosomes, vesicles, and vacuoles [146,147] by preventing the entry of H^+^ into these organelles. BafA also inhibits the fusion of autophagosomes with lysosomes, by disruption of Ca^2+^ gradients implied in this process [148].

Ionophores can also disrupt lysosomal pH, impairing the autophagy process. Tambjamine analogues are anion selective ionophores derived from the naturally occurring tambjamines and induce mitochondrial swelling and autophagy blockade with cytotoxic effects in lung cancer cells and cancer stem cells (CSCs) [149]. Monensin, nigericin, and lasalocid are cation ionophores, but only monensin presents selectivity for lysosomes [150]. Squaramides are synthetic chloride transporters that also induce cell death by apoptosis [151].

On the other hand, the WX8-family comprises five chemical analogs that disrupt the fusion of lysosomes with autophagosomes, lysosomes fission, and sequestration of molecules into the lysosomes without altering their pH. These compounds bind to PIKFYVE phosphoinositide kinase and present potent antitumoral effects on autophagic dependent cells [152]. Vacuolin-1 activates RAB5A blocking the fusion of the autophagosomes with lysosomes, however it also inhibits the fusion of endosomes with lysosomes, resulting in a general endosomal-lysosomal degradation defective [153].

Clomipramine (CM) is a FDA-approved prodrug for the treatment of psychiatric disorders the metabolite of which, desmethylclomipramine (DCMI), impairs autophagic flux blocking lysosomal degradation that sensitizes tumor cells to cancer treatment [154]. DCMI also affects lung CSCs [186]. Additionally, protease inhibitors can also inhibit the lysosomal degradation, such as pepstatin A (aspartyl proteases; cathepsin D and E), Leupeptin [155] and E64d (cysteine proteases; cathepsin B, H, and L) [156]. On the other hand, nanoparticles are usually accumulated into lysosomes by endocytosis internalization, which may cause lysosome impairment [157]. Gold nanoparticles [158] and nanodiamonds have shown to inhibit autophagy by disruption of lysosomal function, which sensitizes tumors to arsenical base therapy [159].

Several studies have suggested that the anti-tumor effects of lysosomal inhibitors may be independent of autophagy inhibition since they also interfere in other cellular mechanisms producing non-autophagy related effects [187,188,189,190,191,192,193,194]. Remarkably, disruption of the lysosomes not only blocks autophagy, but lysosomal permeabilization releases proteases such as cathepsins that are active at cytosolic pH and participate in apoptosis and apoptosis-like and necrosis-like cell death [195,196,197]. Additionally, lysosomes also participate in tumor invasion, hence, these inhibitors have shown to be effective against metastasis [138,198,199,200], targeting cancer stem cells [201], and inducing tumor vessel normalization [202].

As mentioned above, there are efforts to find genetic determinants to sensitivity or resistance to these lysosomal inhibitors. Metastatic cells are more vulnerable to CQ and BafA, suggesting that patients with metastasis could benefit from those treatments [198]. Morgan and coworkers also showed a relationship between the expression of ID4 and metastatic potential. Additionally, overexpression of helicase-like transcription factor (HLTF) seems to be related with the resistance to HCQ, Lys05 and BafA treatment [189] and tumors with the V600E mutation in BRAF (v-Raf murine sarcoma viral oncogene homolog B) present cytoprotective autophagy [203].

## 5. Autophagy Modulation for Tumor Sensitization to Anticancer Therapies

It has been accepted that chemotherapy, as well as radiotherapy, could activate autophagy. This opens the possibility that modulation of autophagy may enhance sensitivity to these cancer treatments [204]. In this section, we highlight some combination therapies that use compounds that target autophagy-inducing sensitization of cancer cells to anticancer therapies.

### 5.1. Autophagy Modulation to Overcome Radio-Resistance

One of the first-line treatments for many types of cancers is radiotherapy. The combination of autophagy inhibitors and radiation therapy has shown improved anti-tumor effects in cancer treatment. For instance, the ATG4B inhibitor NSC185058 [205] and CQ enhance the antitumor effect of radiotherapy in glioblastoma. Furthermore, non-cytotoxic amounts of CQ enhanced the radiation sensitivity in bladder cancer cell lines [206].

The activation of autophagy also radiosensitizes the cells [207] and this effect is increased in the presence of apoptosis inhibitors [208]. Thus, combination therapies based on autophagy stimulation have been formulated to overcome radioresistance in some tumors. For example, YCW1, an optimized HDACI, enhances radiosensitivity in breast cancer cells inducing ER stress and increasing autophagy [209]. Similar results have been reached using a combination of radiotherapy, THC, and cannabidiol in glioma [210] and gossypol in glioblastoma multiforme [211].

As mentioned before, the effect of autophagy after radiation is not uniform in all types of tumors; it can be cytoprotective, non-cytoprotective, or have a cytotoxic effect [212]. Hence, the efforts should be focused on the determination of some markers that could predict the effect of autophagy modulators in combination with radiotherapy. For instance, the expression of p53 has been suggested to be determinant for a radiosensitization effect of CQ [213] and p18-CycE (proteolytic cyclin E fragment) [214]. Nrf2 antioxidant pathway seems to be involved in autophagy-induced radioresistance, which is reversed by 3MA co-treatment [215]. Phosphatase and tensin homolog deleted on chromosome 10 (PTEN) loss is associated with radio and chemoresistance, and activation of autophagy by rapamycin induces cytotoxic autophagy that overcomes radioresistance [216]. 

It should be noted that the models used to analyze these radiosensitization effects are of major importance in order to obtain feasible results. For instance, inhibition of autophagy combined with radiotherapy in immunodeficient mice has a sensitization effect; however, in immunocompetent mice it promotes tumor growth. [217]. This opposite effect is due to the role of autophagy in the immunogenic antitumor response [218]. Therefore, these types of studies should be performed in an immunocompetent context.

### 5.2. Autophagy Modulation to Overcome Chemoresistance

Inhibition of autophagy may sensitize tumor cells to common drugs or may overcome the resistance acquired by those cells to chemotherapeutic agents [219]. In this section, we highlight some of the most recent and relevant findings in combining different antitumor drugs with autophagy inhibitors and some activators.

CQ and HCQ potentiate the cytotoxicity of multiple drugs such as 5-fluorouracil [220], cisplatin [221], and temozolomide [222,223]. Moreover, combination treatment with CQ and trastuzumab completely suppressed tumor growth by >90% in a HER2-positive breast cancer tumor xenograft completely refractory to trastuzumab [224].

Additionally other autophagy inhibitors have shown interesting results; verteporfin increased the potential of gemcitabine in an in vitro model of pancreatic cancer [109]. SBI-0206965 overcame resistance to cisplatin in NSCLC cells [101] and to cabozantinib in metastatic colorectal cancer [225]. The combination of celecoxib, a specific inhibitor of cyclooxygenase-2, with CQ and SAR405 resulted in higher cell death [226], and 3MA also enhanced cell death induced by bortezomib in glioblastoma cell lines [227]. Moreover, the combination of the CQ analog lys05 with the second generation of tyrosin kinase inhibitor, nilotinib, has shown an additive effect in the reduction of the number of leukemia stem cells in CML mouse models [138]. UAMC-2526 potentiates the effect of oxaliplatin in colorectal cancer xenograft mouse model by inhibition of autophagy, and tumors treated with UAMC-2526 also showed a more differentiated phenotype [124].

The combination of autophagy activators and inhibitors together have also been studied; CQ and HCQ potentiate the effect of mTOR inhibitors, such as temsirolimus [228] or everolimus in colorectal cancer cells [229], melanoma [228], and neuroendocrine neoplasms [230], showing inhibition of autophagy as a mechanism to overcome resistance to mTOR inhibitors. In addition, CQ has also demonstrated to improve anticancer effects of vorinostat [231], as well as a combination of everolimus and SAR405 showed synergy [113,232]. These results would make one think that the inhibition of autophagy is a better therapeutic approach because autophagy presents a protective role in these models. However, although to a lesser extent, autophagy activators are also able to overcome chemoresistance. Temsirolimus has demonstrated to potentiate the activity of gemcitabine and cisplatin in bladder cancer cell lines [233] and also decrease the resistance of colon cancer cells to cetuximab [234]. Similarly, curcumin has shown to enhance gefitinib effect on primary gefitinib-resistant small-cell lung cancer cells through an autophagy-dependent synergism [235].

Nevertheless, in some cases, autophagy modulation has shown controversial results in preclinical studies. For instance, it has been shown in vitro that SBI-0206965, an ULK1 inhibitor, induces apoptosis in combination with mTOR inhibitors in A549 cells [100,102]. However, in neuroblastoma cells, SBI-0206965 sensitizes cells to TNF-Related Apoptosis Inducing Ligand (TRAIL) treatment but not to mTOR inhibitors [100] indicating the non-protective role of autophagy in this model. At this point, it would be necessary to use molecular markers that could predict the response of the tumors to autophagy modulators, such as v-Raf murine sarcoma viral oncogene homolog B (BRAF) V600E mutation, which has been associated with protective autophagy [236].

Finally, the development of nanotechnology exploits tumor-targeting therapy directing multiple drugs to the tumor mass, including autophagy inhibitors, which have been encapsulated in different nanocarrier systems to obtain more efficient therapies. On one hand, HCQ nanoencapsulation showed more efficacy than free HCQ [237,238]. On the other, these multiplatform systems allow the combination of autophagy inhibitors with other chemotherapeutic agents [239,240,241], diagnosis system [237], or other therapeutic approaches, such as sonodynamic [242] or photothermal therapy, showing efficient tumor suppression effect [243,244]. Additionally, CQ may be used to promote the accumulation of the drugs in the tumor site; pretreatment with CQ affects macrophages endocytic capacity, which limits nanoparticle accumulation in the liver, reducing liver clearance [245], and improves tumor microcirculation, which promotes co-delivery of antitumoral drugs in tumors [246].

All of the above suggest that modulation of autophagy may be a promising approach to overcome radio and chemoresistance. However, more efforts are needed to predict the response of patients to those treatments.

## 6. Combination Therapy in Clinical Trials

Inhibition and activation of autophagy for cancer treatment has been evaluated in the clinic and clinical trials with autophagy modulators that present results are compiled in Table 3.

CQ and HCQ have been included in 21 and 66 clinical trials for cancer treatment, respectively, 17 of which are currently active and 24 have been completed [6]. In summary, HCQ has limited activity as a single agent, despite the fact that it inhibits autophagy in patients with different early-stage solid tumors [247]. However, the analysis of autophagy in patients with metastatic pancreatic adenocarcinoma showed inconsistent inhibition and no significant therapeutic efficacy [248]. The inhibition of autophagy in combination therapies of HCQ with anticancer agents such as gemcitabine, temozolomide, and bortezomib has been corroborated. However, efficacy results with these and other chemotherapeutic agents, such as erlotinib, were negligible [249] or moderate [250,251]. Additionally, the combination of HCQ with chemotherapy and radiotherapy at the same time inhibited autophagy in 45–66% of patients and this combination did not improve the overall survival rate [252]. Patients with glioblastoma that had received radiotherapy and chemotherapy were treated for 12 months with CQ showing limited results [253]; however, chronic administration of CQ improved the overall survival rate about 50% [254]. Additionally, the combination of radiotherapy with CQ did not improve the overall response rate in patients with brain metastasis [255].

All these results show inconsistency not only at the level of autophagy inhibition but also in the efficacy outcome of this therapy. As mentioned before, the role of autophagy is context dependent, and this has been proven in the clinic. For instance, CQ overcame resistance to vemurafenib, a BRAF inhibitor, in different patients with BRAF^V600E^-mutant brain tumors [203,256]. However, this combination showed no synergistic effect on patients without this mutation [203]. This points out the importance of the identification of patients that are most likely to respond to this combination therapy.

Regarding autophagy activators, (-)-gossypol in combination with cisplatin and etoposide has shown promising results on phase I clinical trial for small cell lung cancer [257]. However, its combination with docetaxel or androgen deprivation therapy have not demonstrated enough efficacy on phase II clinical trial for head and neck cancer [258] or metastatic prostate cancer [259]. Vorinostat in combination with tamoxifen showed moderate response with 40% of response or stable disease in patients with hormone-therapy resistant breast cancer [260].

Finally, the combination of autophagy activators and inhibitors, such as the combination of vorinostat with HCQ has shown promising results [261]. Although rapalog temsirolimus is not powerful enough to be used as a single agent for the treatment of breast and renal carcinoma [262], the combination of HCQ with rapamycin showed a moderate positive response [263]. Moreover, HCQ combined with rapamycin in metronomic chemotherapy showed encouraging results with 40% of partial response and 84% control of disease [264]. Finally, combination of HCQ and temsirolimus also showed stabilization of the disease in 67–74% of the patients with a consistent inhibition of autophagy [265].

## 7. Conclusions

Targeting autophagy for cancer treatment seems to be a promising therapeutic strategy, although significant challenges remain to be addressed in order to improve the therapeutic results obtained in the clinic. In particular, since the outcome of modulating autophagy depends on the tumor context, it must be carefully defined which patients would benefit from which treatment before starting any therapeutic intervention. 

Autophagy suppresses tumor initiation in healthy tissues, showing a cytoprotective role. Hence, the modulation of autophagy through autophagy activators might be beneficial in patients with an increased risk of developing cancers. On the other hand, once the tumor is formed, activating autophagy will induce ACD in some tumors, provoking their reduction. This strategy may be especially relevant in apoptosis-resistant tumors. Nevertheless, autophagy has a protective role in other tumors, especially those called “autophagy-dependent”; hence, the inhibition of autophagy would induce therapeutic effects in those patients. Indeed, inhibition of autophagy at early or late stages leads to different consequences; prevention of the autophagosome formation may neutralize the protective role of autophagy, sensitizing cells to chemotherapeutic agents, being a good strategy for combination regimens. Conversely, lysosomotropic agents produce autophagy vacuoles accumulation, leading to cellular stress and a consequent cytotoxic effect, being able to reduce the tumor in single therapy.

This review underscore the double-edged-sword role of autophagy in cancer; hence, a deeper understanding on how autophagy affects cancer progression, the search of appropriate biomarkers to identify the responder patient population to a defined autophagy modulator, and clear and suitable pharmacodynamic markers to monitor patients’ responses are eagerly needed to improve success in clinical studies with autophagy modulators.

## Figures and Tables

**Table 1:** Autophagy activators.

Mechanism of Action/Type	Name	Structure	Number in Figure 1	Refs.
mTOR Inhibitors	Rapacmycin	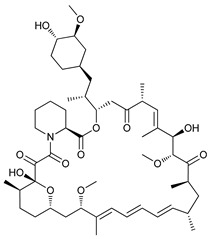	1	[43,44,45,46]
	Temsirolimus (CCI779)	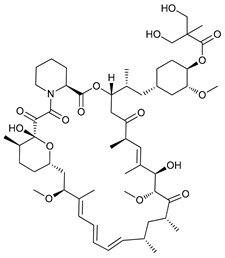	2	[47,48]
	Everolimus (RAD001)	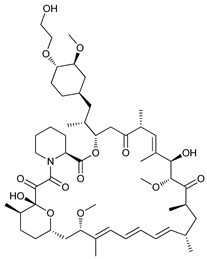	3	[49,50]
	AZD8055	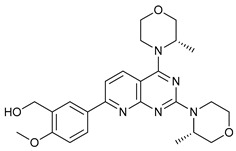	4	[51,52]
BH3 Mimetics	(-)-gossypol (AT-101)	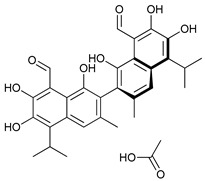	5	[53,54,55,56]
	Obatoclax (GX15-070)	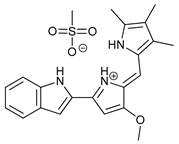	6	[57,58,59]
	ABT-737	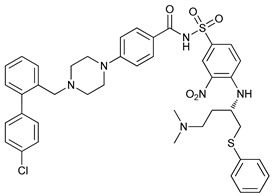	7	[60]
Cannabinoids	Δ9-Tetrahydrocannabinol (THC)	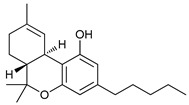	8	[61,62,63]
	JWH-015	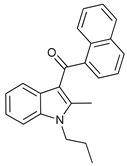	9	[64]
Histone Deacetylase Inhibitors	Suberoylanilide hydroxamic acid (SAHA, Vorinostat)	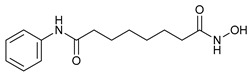	10	[65]
	MHY2256	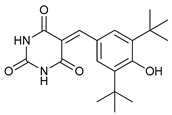	11	[66]
Natural Products	Betulinic acid	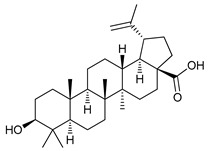	12	[67]
	Resveratrol	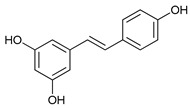	13	[68]
	δ-Tocotrienol	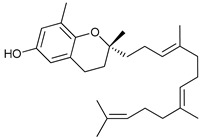	14	[69]
	Curcumin	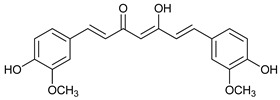	15	[70]
Others	Lapatinib	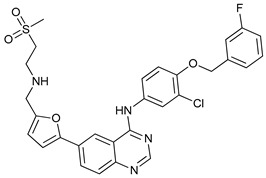	16	[71,72]
	APO866	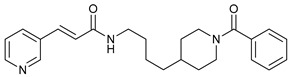	17	[73]

**Table 2:** Autophagy inhibitors.

Mechanism of Action		Name	Structure	Number in Figure 1	Refs.
ULK Inhibitors		Compound 6	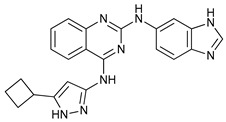	1	[96]
		MRT68921	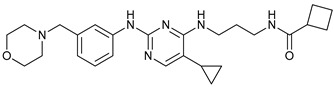	2	[97,98]
		MRT67307	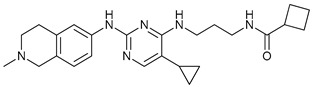	3	[97,98]
		SBI-0206965	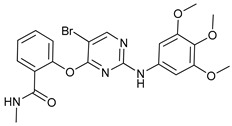	4	[99,100,101,102]
		ULK-100	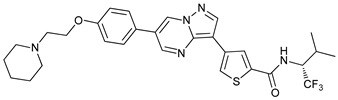	5	[103]
		ULK-101	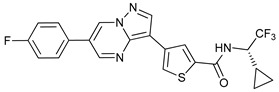	6	[103]
Pan PI3k Inhibitors		3MA	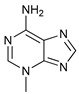	7	[104,105,106]
		3 MA derivatives	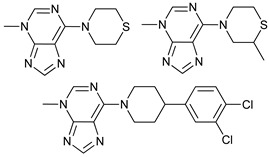	8	[107]
		Wortmannin	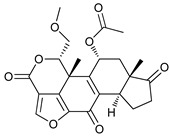	9	[108,109]
		LY294002	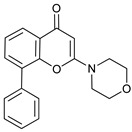	10	[110]
		SF1126	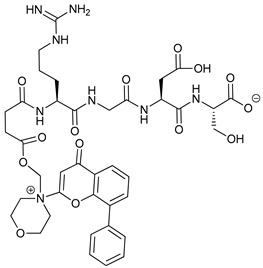	11	[111,112]
		PI103	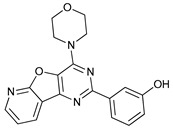	12	[113]
		KU55933	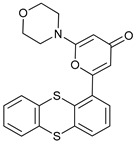	13	[114]
		Gö6976	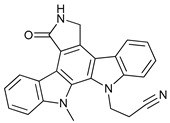	14	[114]
		GSK1059615	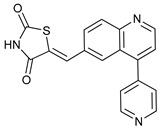	15	[115,116]
VPS34 (PI3KC3) Inhibitors		SAR405	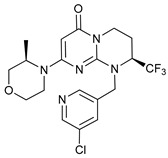	16	[113]
		VPS34-IN1	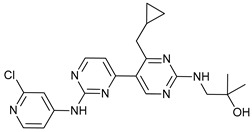	17	[117]
		PIK-III	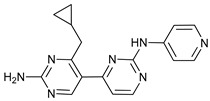	18	[118]
		Compound 31	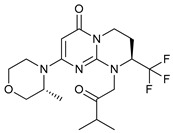	19	[119]
		Spautin-1	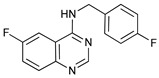	20	[120]
ATG Inhibitors		ATG7 inhibitor	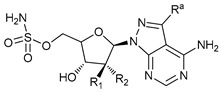	21	WO2018/089786
		ATG7 inhibitor, miR154	UAGGUUAUCCGUGUUGCCUUCG	22	[121]
		NSC185058	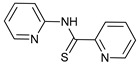	23	[122]
		Tioconazol	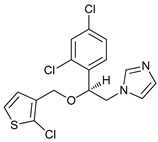	24	[123]
		UAMC-2526	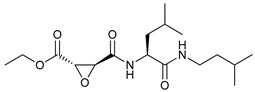	25	[124]
		LV320	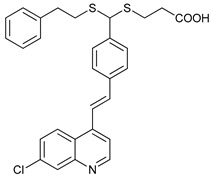	26	[125]
		S130	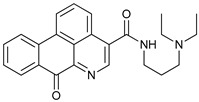	27	[126]
		FMK-9a	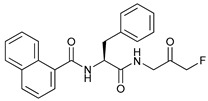	28	[127,128,129]
Autophagy Formation		Verteporfin	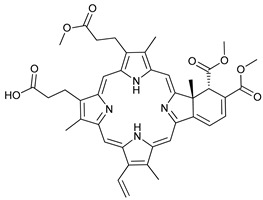	29	[130,131,132,133]
Lysosome Inhibitors	Lysosomotropic Agents	Chloroquine	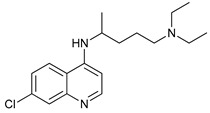	30	[134,135]
		Hydroxychloroquine	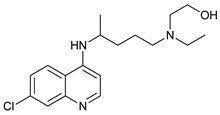	31	[136]
		Lys05	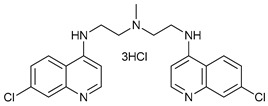	32	[137,138]
		DQ661	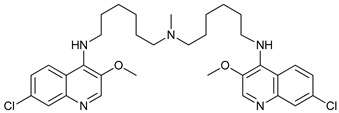	33	[139]
		VATG-027	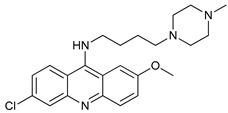	34	[140]
		Mefloquine	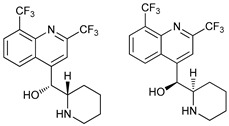	35	[141,142]
		Ganoderma lucidum polysaccharide (GLP)		36	[143,144,145]
	Vacuolar H^+^ ATPase Inhibitors	Bafilomycin A1	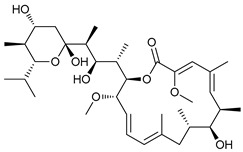	37	[146,147,148]
	Ionophores	Tambjamines	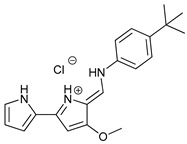	38	[149]
		Monensin	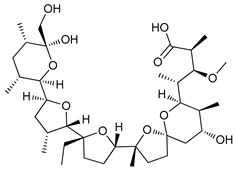	39	[150]
		Squaramides	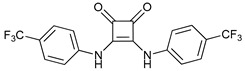	40	[151]
	Inhibition of Autophagosome-Lysosome Fusion	WX8 family	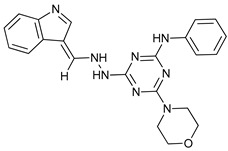	41	[152]
		Vacuolin-1	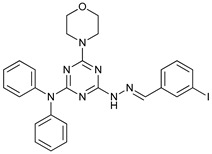	42	[153]
		Desmethylclomipramine	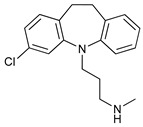	43	[154]
	Acid Protease Inhibitors	Pepstatin A	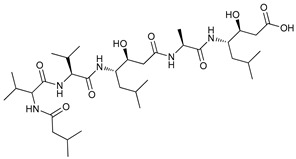	44	[155]
		Leupeptin	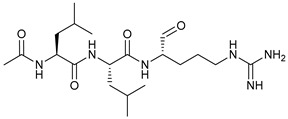	45	[155]
		E64d	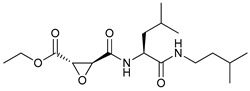	46	[156]
	Others	Nanoparticles		47	[157,158,159]

**Table 3:** Clinical trials results with autophagy modulators. Autophagy modulator column has been added.

Clinicaltrials.Gov ID	Treatment (Dose Per Day)	Autophagy Modulator	Condition	Study Phase	Result	Refs.
NCT01273805	HCQ (1200 mg)	Inhibitor	Metastatic Pancreatic cancer	II	Lack of efficacy	[248]
	HCQ (800 mg)	Inhibitor	Early stage solid tumors	I	Autophagy inhibition, apoptosis	[247]
NCT00771056	HCQ (400 mg)	Inhibitor	B-cell chronic lymphocytic leukemia	II	50% efficacy. No adverse events	
	HCQ (1200 mg) + bortezomib	Inhibitor	Myeloma	I	Autophagy Inhibition. Moderate response	[251]
NCT00786682	HCQ (400 mg) + docetaxel	Inhibitor	Prostate cancer	II	Terminated; lack of efficacy	
NCT01649947	HCQ (400 mg) + Paclitaxel + Carboplatin + Bevacizumab	Inhibitor	NSCLC	II	Evaluation of Bevacizumab addition to the drug cocktail	
NCT01026844	HCQ (1000 mg) + Erlotinib	Inhibitor	Advanced NSCLC	I	Safe but low efficacy	[249]
NCT00977470	HCQ (1000 mg) + Erlotinib	Inhibitor	Advanced NSCLC	II	Not completed; lack of efficacy	
NCT01978184	HCQ (1200 mg) + Gemcitabine + paclitaxel	Inhibitor	Pancreatic cancer	II	Moderate results	[266,267]
NCT01128296	HCQ (1200 mg) + Gemcitabine	Inhibitor	Pancreatic cancer (stage IIb III)	I–II	65% Autophagy inhibition	[268]
NCT00486603	HCQ (600 mg) + Temozolomide + radiation	Inhibitor	Glioblastoma multiforme	I–II	Autophagy inhibition in 45–66%. 70% affected by serious adverse effects. Lack of efficacy	[252]
	HCQ (1200 mg) + Temozolomide	Inhibitor	Advanced solid tumors and melanoma	I	Autophagy inhibition. Moderate results	[250]
NCT01842594	HCQ (400 mg) + Rapamycin	Inhibitor + Inducer	Sarcoma	II	Terminated; 60% partial response.	[269]
NCT01687179	HCQ (400 mg) + Rapamycin	Inhibitor + Inducer	Lymphaglioleiomyomatosis	I	Well tolerated. Limited response	[263,270,271]
	HCQ (1200 mg) + Temsirolimus	Inhibitor + Inducer	Advanced solid tumors and melanoma	I	Well tolerated, autophagy inhibition. Moderate response	[265]
	HCQ (600 mg) + Vorinostat	Inhibitor + Inducer	Advanced solid tumors	I	Well tolerated, moderate response	[261]
	HCQ (400 mg) + Rapamycin + metronomic conventional chemotherapy	Inhibitor + Inducer	Solid tumors	I	Encouraging results	[264]
	CQ (150 mg)	Inhibitor	GBM	II	Encouraging results	[185]
	CQ (150 mg) + Carmustine	Inhibitor	GBM		Limited response	[253]
	CQ (250 mg) + radiotherapy	Inhibitor	GBM	Pilot	Encouraging results (5 patients)	[272]
NCT01894633	CQ (150 mg) + radiotherapy	Inhibitor	Brain metastais	II	Limited response	[255]
	CQ (250 mg) + radiotherapy	Inhibitor	Brain metastasis	Pilot	Well tolerated	[273]
	CQ (150 mg) + vemurafenib	Inhibitor	BRAF^V600E^ Brain tumor		Encouraging results (6 patients)	[203,256]
NCT00365599	Vorinostat (400 mg) + Tamoxifen	Inducer	hormone-therapy resistant breast cancer	II	Moderate response	[260]
	(-)-gossypol (80 mg) + cisplatin + etoposide	Inducer	SCLC	I	Encouraging results	[257]
	(-)-gossypol (80 mg) + docetaxel	Inducer	Head and neck cancer	II	Lack of efficacy	[258]
NCT00666666	(-)-gossypol (20 mg) + androgen deprivation therapy	Inducer	metastatic prostate cancer	II	Lack of efficacy	[259]

## Abbreviations

ACD	autophagic cell death
AMPK	AMP-activated protein kinase
ATG	Autophagy-related proteins
ATG16L1	Autophagy-related 16-like protein 1
BafA	Bafilomycin A
BH3	Bcl-2 Homology 3
BRAF	v-Raf murine sarcoma viral oncogene homolog B
CMA	Chaperone Mediated Autophagy
CB_1 y 2_	Cannabinoid receptor 1 y 2
CCI779	Cell Cycle Inhibitor 779
CML	Chronic Myeloid Leukemia
CQ	Chloroquine
CSC	Cancer stem cell
DCMI	Desmethylclomipramine
DQ	Dimeric quinacrine
EGFR	Epidermal growth factor receptors
ER	Endoplasmic reticulum
FIP200	RB1-inducible coiled-coil protein 1
FKBP12	FK506-binding protein 12
GABARAP	γ-aminobutiric acid receptor-associated proteins
HCQ	Hydroxychloroquine
HDAC	Histone deacetylase
HDACIs	Histone deacetylase inhibitors
LAMP2	Lysosome-associated membrane protein 2
LC3	Microtubule-associated protein light chain 3
MQ	Mefloquine
mTORC1	Mammalian target of rapamycin complex 1
mTORC2	Mammalian target of rapamycin complex 2
PE	Phosphatidylethanolamine
PI3K	Phosphoinositide 3-kinase
PI3KC1	The class I phosphatidylinositol 3-kinase
PI3KC3	The class III phosphatidylinositol 3-kinase
PtdIns3P	Phosphatidylinositol 3-phosphate
ROS	Reactive oxygen species
SAHA	Suberoylanilide hydroxamic acid
SNAP29	Synaptosomal-associated protein 29
SNARE	Soluble N-ethylmaleimide-sensitive factor activating protein receptor
STX17	Syntaxin 17
THC	Δ^9^-Tetrahydrocannabinol
TK	Tyrosin Kinase
TOR	Target of rapamycin
TRB3	Telomere repeat binding factor 3
ULK	(Unc)-51–Like Kinase proteins
VPS15	Vacuolar Protein Sorting 15
VPS34	Vacuolar Protein Sorting 34
